# Role of palliative care in fetal neurological consultations: Guiding through uncertainty and hope

**DOI:** 10.3389/fped.2023.1205543

**Published:** 2023-06-02

**Authors:** DonnaMaria E. Cortezzo, Marissa Vawter-Lee, Abdullah Shoaib, Charu Venkatesan

**Affiliations:** ^1^ Cincinnati Children's Hospital Medical Center, Division of Neonatal and Pulmonary Biology, Cincinnati, OH, United States; ^2^ Cincinnati Children's Hospital Medical Center, Division of Pain and Palliative Medicine, Cincinnati, OH, United States; ^3^ Department of Pediatrics, University of Cincinnati College of Medicine, Cincinnati, OH, United States; ^4^ Department of Anesthesiology, University of Cincinnati College of Medicine, Cincinnati, OH, United States; ^5^ Cincinnati Children's Hospital Medical Center, Division of Neurology, Cincinnati, OH, United States

**Keywords:** counseling, congenital malformation, prenatal, pregnancy, brain, perinatal palliative care, uncertain prognosis

## Abstract

Fetal neurology is a rapidly evolving and expanding field. Discussions about diagnosis, prognosis, treatment options, and goals of care often begin in the antenatal period. However, there are inherent challenges to fetal counseling of neurological diagnoses due to limitations of fetal imaging, prognostic uncertainty, and variability in neurodevelopmental outcomes. In the midst of uncertainty, families are challenged with preparing a care plan for their baby while simultaneously experiencing profound grief. The paradigms of perinatal palliative care can assist with the grieving process and help frame diagnostic testing and complex decision-making within the context of a family's spiritual, cultural, and social belief system. This ultimately leads to a shared decision-making process and value driven medical care. While perinatal palliative care programs have expanded, many families faced with such diagnoses never meet with a palliative care team prior to delivery. Moreover, there is significant variability in the availability of palliative care services throughout the country. Using an illustrative vignette of a patient with a prenatally diagnosed encephalocele, this review aims to provide a basic framework of perinatal palliative care for fetal neurology diagnoses that emphasizes 1) importance of clear, consistent, and transparent communication among all subspecialists and families, 2) creation of a palliative care birth plan, 3) importance of consistent care providers and longitudinal points of contact prenatally and post-delivery, 4) close communication between the prenatal and post-natal providers to allow for optimal continuity of care, and 5) recognize that information, care plans, and goals of care often evolve over time.

## Introduction

Perinatal palliative care was historically considered for families and providers only when a life-limiting diagnosis was made during the perinatal or neonatal period (1–10). However, with advances in technology and treatment options, there is an increase in prenatally identified neurological disorders carrying uncertain or potentially life-limiting prognoses (11–14). Recognizing that these families face many of the same challenges and decisions during pregnancy and after delivery as those with a lethal diagnosis, the scope of perinatal palliative care has expanded so that these families can also benefit from the paradigms of palliative care.

For example, encephaloceles, which are congenital malformations characterized by herniation of intracranial contents through a defect in the skull, can result in a wide range of outcomes in terms or morbidity or mortality (15–17). The mortality rates in the neonatal period, even with invasive medical and surgical treatment, are as high as 33.3% (15, 18–20). Most of the deaths occur in the first days of life and up to 90% of those who undergo surgery survive the first years of life (17, 21). There is variability in morbidity and neurodevelopmental impairments reported with some studies reporting >50% of individuals having significant impairments that preclude living independently and others reporting 40% as having no neurodevelopmental impairments during early follow up (15, 22–28). A recent study of 102 patients noted that 14.9% had mild to moderate delays and 18.1% had severe delays (17). The presence of neural tissue in the defect was associated with neurodevelopmental disabilities. With a wide range in prognoses and imprecise predictors of outcomes, prenatal counseling is imperative to support families and help them navigate goals of care, recognizing that goals may shift over time.

## Case

A 19 year-old G1P0 woman at 21 5/7 weeks gestation with a fetus with a large encephalocele was referred for evaluation to a fetal care center. Fetal magnetic resonance imagining (MRI) was remarkable for a large occipital encephalocele containing most of the occipital and parietal lobes. The posterior fossa was small with herniation of brainstem and cerebellar tissue. The family met with maternal-fetal medicine, neurosurgery, neonatology, and neurology and was extensively counselled about the spectrum of developmental outcomes, including epilepsy and motor, cognitive, language, social and visual impairment. Given brainstem herniation and presumed functional compromise, possible difficulties with breathing independently and feeding orally were discussed. Care options including termination of pregnancy, medical interventions, comfort measures and potential decisions related to surgeries and respiratory support were introduced. After learning the prognosis and spectrum of outcomes, the family was worried he would have an unacceptable quality of life. They expressed interest in learning more about comfort measures only approach to care.

### Discussion

Herniation of posterior fossa contents including brainstem should raise concern about compromise of brainstem functions including breathing and feeding independently after birth. It is important to clearly communicate with families and raise the possibility of neonatal death without medical intervention to support these functions. However, given the uncertainty, explaining that the baby may be able to breathe with minimal or no support is also important. Providers often use terms such as “lethal” or portray the prognosis of various diagnoses as “grim” instead of clearly articulating the spectrum of outcomes (29–32). Families may then feel unprepared and can develop mistrust of the medical community. Providing concrete details about outcome allows families to know the breadth of possibilities in the moments after birth. It is helpful to guide families in chronological progression starting with expectations of possible functional impairments after birth and means to address the various clinical presentations. For example, if the ability to feed orally is compromised, possibilities such as partial or full support *via* nasogastric tube with inclusion of details such as maternal breast milk or formula per family preference are important points of awareness. Attention to language and terms used is crucial. For example, clinicians often say “significant risk” when there is certainty of an adverse outcome. Families can interpret that to mean there is a possibility of normal outcome. Similarly “developmental delay” is used to convey “developmental deficit.” Families will interpret “delay” to mean that the child “will catch up” as opposed to a permanent deficit in function. It is also important to discuss, in the context of the spectrum of outcomes, the implications for the pregnant individual and all options for the pregnancy. The pregnant individual is at increased risk for medical complications, hospitalizations, and invasive procedures (33, 34).

For the clinician, it is tempting to relay all the medical information in the form of a monologue so that families fully hear the diagnosis and prognosis. This, however, does not mean they are truly informed. Clinicians must attend to the emotional frame of mind of families and recognize that they may not be in a receptive or cognitive state at that moment to process all the medical information (35, 36). Frequent pauses and check-ins can help gauge their understanding of the information and their emotional state. It may be necessary to pause the discussion and have follow-up visits when they can receive and process more medical information. Families may feel overwhelmed in a large team meeting with multiple providers. It may be beneficial for Neurology or Palliative Care to meet separately with the family to allow time to discuss the prognosis and to begin exploring values, goals, and views of quality of life.

## Case continues

Given the uncertain prognosis and medically complex decision-making, the family was referred to a perinatal hospice program. They continued to navigate their goals of care and focus on memory-making through the remainder of the pregnancy. They also developed a detailed birth plan expressing their hopes and wishes. After multiple discussions about prognosis, values, and quality of life, they wished for a comfort measures only approach to care. The medical providers and family had copies of the birth plan. The entire care team was aware of goals of care leading up to the time of delivery.

### Discussion

When families learn of a potentially life-limiting diagnosis or a diagnosis with an uncertain prognosis, they often need time to process the information and incorporate it into decision-making (37). Medical information should be framed in a manner that considers families' cultural and ethical values, views on quality of life and disability, their social structure and their hopes (31, 38–40). Conversations should occur in a manner that is sensitive, nonjudgmental and allows for exploring values and goals of care. These discussions and decisions, albeit difficult, give families a sense of control (41).

Families appreciate continuity of care and the opportunity to share about themselves and their journey (5, 10, 31). Clear and consistent information from a multidisciplinary team makes families feel supported and assured that they have the full breadth of information necessary to make complex medical decisions (5, 42–44). Unfortunately, communication is often lacking and information, at times, is given in a rushed or insensitive manner. When perinatal palliative care teams are available, they help provide continuity and aid in communication and navigating goals of care (45, 46). Beyond that, in the midst of uncertainty, they provide spiritual and psychological support to the family, focus on memory-making, and celebrate moments during the pregnancy. In this case, after enrolling in perinatal hospice, the family was able to make memories such as heartbeat recording and plan for the future while grieving the pregnancy and birth they had expected. They also received spiritual support and counseling.

While there are clear benefits, many families never meet with a palliative care provider. There is variation in the availability of pediatric palliative care and the services they provide (47–50). Thus, other subspecialists are tasked with providing support, navigating goals of care, and birth planning. There are many tools to help providers navigate these discussions. REMAP is one example of a tool to help explore goals of care (35, 36). With this model, providers are encouraged to Reframe the medical understanding, Expect emotion, Map out the family's values system, Align with their values, and Plan for the future. Table 1 outlines how a provider may use REMAP in the context of a fetal neurological diagnosis with an uncertain prognosis.

**Table 1:** Exploring goals of care for families facing and fetal neurological diagnosis with an uncertain prognosis (modified REMAP framework (1, 36).

Reframe medical understanding and the range of prognoses
• Help family explore “What does this really mean?” • Acknowledge uncertainly in the prognosis and the range of medical implications • Balance “hope and reality” in the face of uncertainty • “*While we do not know exactly what this means for your son, we know this is a very serious diagnosis and I am worried that he*…”
Expect emotion
• Ask open ended questions • Validate and respond to emotion • Respond with empathy • “*I see that you are very worried. Can you tell me what you are worried about*?”
Map out value system family uses to makes decisions.
• Inquire about faith, spirituality, religion • Learn how they as a family make decisions • Help put their values in context of the current situation • “*Given what we have learned about your son, can we think about what is most important for you as his parents*?”
Align with values and hopes
• Reflect and summarize what you are hearing • Seek clarification and verification • Align yourself with their values • “*It sounds like what is most important to you as parents is (spending time, making sure he is comfortable, not losing hope)…*
Propose a plan
• Suggest how identified goals may be achievable • Suggest treatments and paths that match their values • “*Given what we know about your son and what you said is most important as parents, it might be helpful if we create a plan that focuses on (time, comfort, better understanding diagnosis, etc.) for baby's care after delivery*.”

It is important to communicate what is learned while exploring goals of care. A detailed birth plan that communicates the diagnosis, hopes, and plan for the mother and baby at the time of and after delivery is helpful for both the family and the medical team. The process of birth planning can be meaningful and therapeutic to families (1). It often culminates in a physical document that expresses their wishes so that the entire care team has an understanding of their goals and care can be seamless throughout transitions in providers and hospitals (46). It is also important to note that providers and parents may have different goals with a birth plan. Parents desire clear communication and their primary focus centers around the time with their baby after birth, while providers hope for details about the family's understanding, any ambiguities in the care plan or decision points, and details about the medical plan (1, 46). It is important that prior to creating these plans, individuals with expertise on obstetrics management, such as the higher likelihood of a cesarean section due to breach presentation, failure to progress, or macrocephaly, share this knowledge with the family so that their expectations and subsequent plans align. The birth plan should ideally be viewed as an advance care planning document that describes the medical care that is desired, the values, and wishes (46). The birth plan should include a wide range of information important for providing care such as (1) name of the baby and parents, (2) diagnosis and pertinent medical information, (3) contact information for medical providers and support people, (4) wishes for labor and delivery (including wishes for fetal monitoring and mode of delivery), (5) wishes for the medical care of the baby including decision points, (6) wishes for memory-making and support, (7) plan of care if the baby survives, (8) plan of care if baby dies, and (9) any additional requests from family (46). The birth plan should be located in the electronic medical record and in multiple physical locations so all providers have access to it. Providers involved in birth planning should create a detailed note as a communication tool in the mother's chart with additional medical details important to the care team (46). It should include (1) family members present at the meeting, (2) maternal and obstetric history, (3) results of fetal testing and imaging, (4) name of the subspecialists involved in the medical care, (5) social history (family support, stressors, coping, involvement in the decision-making process), (6) impressions/counseling/plan (what the family has been told to expect regarding morbidity, mortality, anticipated medical challenges, treatment options, and how that is expected to change the disease trajectory) and (7) goals of care (wishes of the family, including details if there are conflicting goals) (46). These measures help ensure that care is consistent and the focus remains on value driven medical care.

## Case continued

After delivery at term, the baby was stable on room air, orally feeding, and stayed with his mother. He was microcephalic with a large occipital encephalocele covered by a thin membrane. He was discharged home at several days of life with a do not resuscitate (DNR) order, comfort medications, and the support of hospice. There were multiple home visits in the first weeks of life to address protecting the encephalocele, pain, and potential for infection, apnea, seizures, and feeding difficulties. Family affirmed that they did not wish to pursue surgery but that they would want “simple” infections treated. At two weeks, he was referred to a pediatrician with close ties to the hospice team for his routine care as he was eating by mouth, breathing effectively on room air, and not showing signs of decline. At several months, he presented to the emergency room with purulent discharge from the encephalocele. During initial conversations in the emergency room with the palliative care team and primary care physician, family wished for him to receive intravenous antibiotics and to be a full code. With continued discussions about goals of care, family expressed their desire for hydration and antibiotics. However, they did not want respiratory interventions or cardiopulmonary resuscitation.

### Discussion

Because the family was prepared for the spectrum of outcomes, they understood that despite not requiring invasive medical interventions, the overall prognosis remained the same. Families can have conflicting feelings about their child's best interests when they exceed expectations irrespective of the poor neurodevelopmental prognosis. It is important to continue open conversations about parental hopes, fears, and goals so that the focus remains on their values and views of quality of life and how medical interventions will or will not achieve them. Discussions, when possible, should be proactive. Weaver et al. suggest considerations when supporting parental decision-making in the context of a diagnosis of trisomy 13 or 18 (51). These principles can be applied to any medically complex diagnosis that carries an uncertain or potentially life-limiting prognosis. We should consider how involved the family wishes to be in making decisions, how they make decisions, what type of information is important, and how they best process information (e.g., visual/written/big picture/details). In addition to assessing families' understanding of the medical diagnosis and treatment options, it is important for providers to be supportive and understand how the medical information is important in the context of the family's individual circumstances. Clinicians must be intentional about the timing of these conversations as it is often necessary to have multiple discussions before making decisions. In these conversations, the focus should be on exploring the family's goals, values, hopes, and fears. What does quality of life mean to them and has that changed over time? What are their goals in the context of the diagnosis? It can be helpful to validate their chosen care path and prepare them for medical decisions they might need to make in the future and anticipated comorbidities. It is important to provide consistent care with familiar providers, emotional support, and the recognition that support and conversations will continue as the medical team walks along the journey with them.

## Case continued

Over the next few months, he had numerous episodes of cerebrospinal fluid drainage from the encephalocele, skin dehiscence, and infections treated with oral antibiotics. Parents understood that he could die from bacterial meningitis and declined surgical intervention. He was followed closely by his pediatrician, home hospice team, and early intervention programs. At 2 years, his pediatrician and hospice team had further discussions around goals of care. The family felt he exceeded their expectations. The encephalocele was frequently infected, negatively impacting his daily care, and limiting his mobility, development, and ability to participate in therapies. They wished for him to remain a DNR and were open to discussing surgical options with neurosurgery. Over the next 6 months there were multiple meetings with the family, neurosurgery, plastics, palliative care, and primary care. The repair, reconstruction, potential need for a shunt, and the risks for infection, hemorrhage, stroke and death were discussed as well as forgoing surgical interventions. The family opted to make him a full code and proceed with surgery. They had detailed discussions about goals of care and treatment plans if surgery went well or if there were significant complications. He underwent resection of the encephalocele and subsequent ventriculoperitoneal shunt placement. At several years of age, he was discharged from hospice and was followed by his pediatrician and numerous subspecialists for epilepsy, cortical visual impairment, global developmental impairments, spastic cerebral palsy, and incontinence. He thrived at home as he interacted with his family and environment, sat independently, said several words, followed simple commands, and was in school.

## Discussion/conclusion

Perinatal palliative care is a comprehensive approach to care that allows medical teams to attend to the total needs of a family when a pregnancy is faced with a serious or potentially life-limiting diagnosis (8, 9, 52–56). As families process the diagnosis, expectations are adjusted. A time that is typically filled with joy and anticipation is now filled with uncertainty, grief, hope, and complex medical decision-making (9, 56–59). With the support of a care team, the family can start the bereavement process while making beautiful memories, reflecting on what quality of life means to them, how they wish to spend time with their baby and what type of medical care they wish to be provided (3, 4, 52, 60). This total care also improves communication with the medical team, often through meetings and birth plans, and ensures everyone understands the plan for the remainder of the pregnancy, the birth, and beyond (1, 54, 61). Parents have expressed that this process gives them a sense of control and allows them to spend precious moments parenting as they had envisioned (3, 10, 61–68). Regardless of the specific model of perinatal palliative care provided, the benefits of having a dedicated care team walk this journey with the family is important and as a result, there has been a call for perinatal palliative care services (6, 7, 10, 53, 54, 69–72). Likely, providers from multiple subspecialties will be tasked with incorporating the paradigms of palliative care and navigating goals of care in their interactions with families. It is important that families have a dedicated care team who forms connections with them and understands their values and decision-making process (72). This ensures that conversations continue and that interventions and therapies are focused on the ultimate goals of the family, realizing that these goals may change over time. Communication of these conversations among the multiple providers involved is even more important when the prognosis is uncertain (73). In situations where a neonate is not actively dying and has complex medical needs, it is imperative that care is established with a provider who can attend to the multiple complex medical needs of the family and can engage in challenging conversations (74, 75). Table 2 outlines a journey map for providers as they continue to support the family during transitions and different phases of care. Through education and a continued, close partnership among palliative care, subspecialists, and the primary care provider, the patient and family can receive multidisciplinary care, feel supported and ultimately navigate medical decisions in a manner that ensures that quality of life remains a focus of care.

**Table 2:** Journey Map across phases of care.

	Shortly after diagnosis	Remainder of the pregnancy	Delivery and birth	Neonatal care	Post-natal care
**Key Take Home Points**	• Expect grief and anxiety • Discuss uncertainty • Provide consistent, clear information • Provide written information • Give time to process information • Offer follow up	• Additional Information can be powerful or cause more uncertainty • Reassure them • Listen (what information are they are hoping to gain) • Explore hopes/fears/values • Discuss options/plans • A decision does not need to be made immediately/evolving plan • Develop birth plan • Establish a clear follow up plan • Consistent care team	• Ensure all are aware of the situation, wishes, birth plan • Focus on bonding • Prepare the family • Honest discussions if the baby is doing better or worse than expected (does it change the prognosis?) • Prepare for discharge or ICU • Ensure all subspecialists and primary care providers are involved	• Frequent, clear, consistent communication • Shared decision-making • Support parents in their role as parents	• Multidisciplinary care • Anticipate decision points and transitions in care (discuss early) • Consistent care team • Consistent follow up • Ensure the family has the support they need • Realize that goals evolve and can change over time • Align with the family

## Data Availability

The original contributions presented in the study are included in the article, further inquiries can be directed to the corresponding author.
